# Understanding neuropathic pain: the role of neurophysiological tests in unveiling underlying mechanisms

**DOI:** 10.1186/s44158-024-00212-z

**Published:** 2024-11-18

**Authors:** Caterina Maria Leone, Andrea Truini

**Affiliations:** Department of Human Neuroscience, Sapienza University, Rome, Italy

**Keywords:** Chronic pain, Neuropathic pain, Central sensitization, Neurophysiology

## Abstract

Neuropathic pain, arising from lesions of the somatosensory nervous system, presents with diverse symptoms including ongoing pain, paroxysmal pain, and provoked pain, usually accompanied by sensory deficits. Understanding the pathophysiological mechanisms behind these symptoms is crucial for targeted treatment strategies. Neurophysiological techniques such as nerve conduction studies, reflexes, and evoked potentials help elucidate these mechanisms by assessing large myelinated non-nociceptive fibres and small nociceptive fibres. This argumentative review highlights the importance of tailored neurophysiological assessments for improving our understanding of the pathophysiological mechanisms behind neuropathic pain symptoms.

## Introduction

**Fig. 1:**
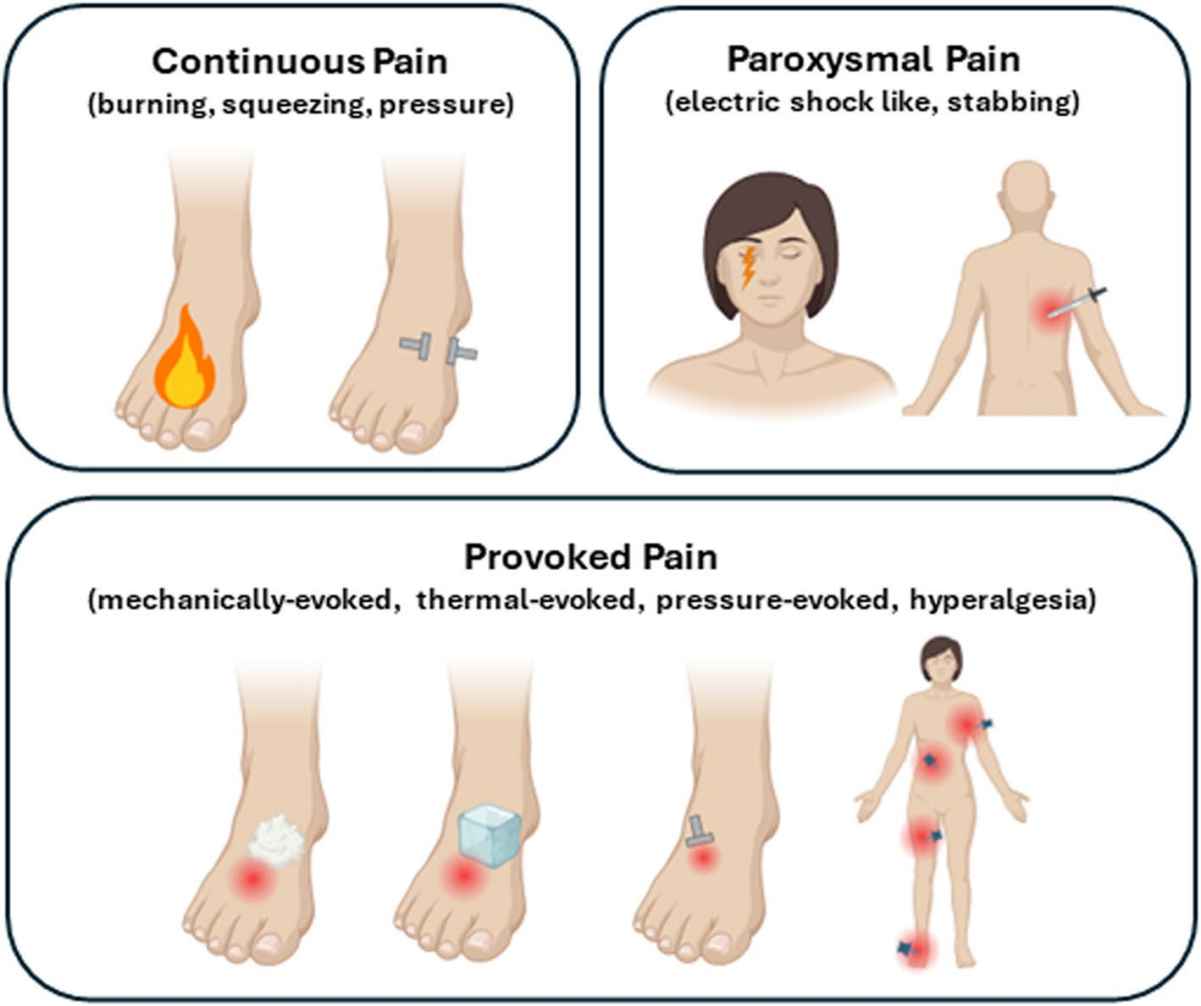
Symptoms of neuropathic pain (created with biorender)

Neuropathic pain is caused by a lesion or disease of the somatosensory nervous system (https://www.iasp-pain.org/Education/Content.aspx?ItemNumber=1698#Neuropathicpain), either in the peripheral or central nervous system. It commonly manifests with a complex combination of distinct symptoms and signs, defined as sensory profile [7]. The more frequent symptoms include ongoing pain (burning, squeezing, pressure), paroxysmal pain (electric shocks, stabbing), and provoked pain (brush-evoked, pressure-evoked, cold-evoked, hyperalgesia) [4] (Fig.1), commonly associated with positive symptoms (paraesthesia) and negative signs (hypoesthesia, hypoalgesia).

Exemplary conditions of neuropathic pain include postherpetic neuralgia, trigeminal neuralgia, painful peripheral neuropathies, and central post-stroke pain.

Several clinical and neurophysiological observations suggest that the different symptoms of neuropathic pain arise through distinct pathophysiological mechanisms [72]. These mechanisms do not depend on the aetiology of the disease, as the same mechanism can be found in different diseases. The peculiarity of the pathophysiological mechanisms behind the different neuropathic pain symptoms suggests that distinct therapies are needed for the treatment of neuropathic pain, targeting the specific pathophysiological mechanism. This underscores the importance of identifying underlying pain mechanisms in patients suffering from neuropathic pain to provide a more effective and specific mechanism-based treatment approach [7]. As an example, previous studies found that the sodium channel blocker oxcarbazepine was more effective in patients with the ‘irritable nociceptor’ phenotype, characterized by hypersensitivity to stimuli and preserved small-fibre function [21]. Conversely, patients with allodynia responded better to botulinum toxin A, and fewer thermal deficits were associated with greater treatment efficacy [5].

**Fig. 2:**
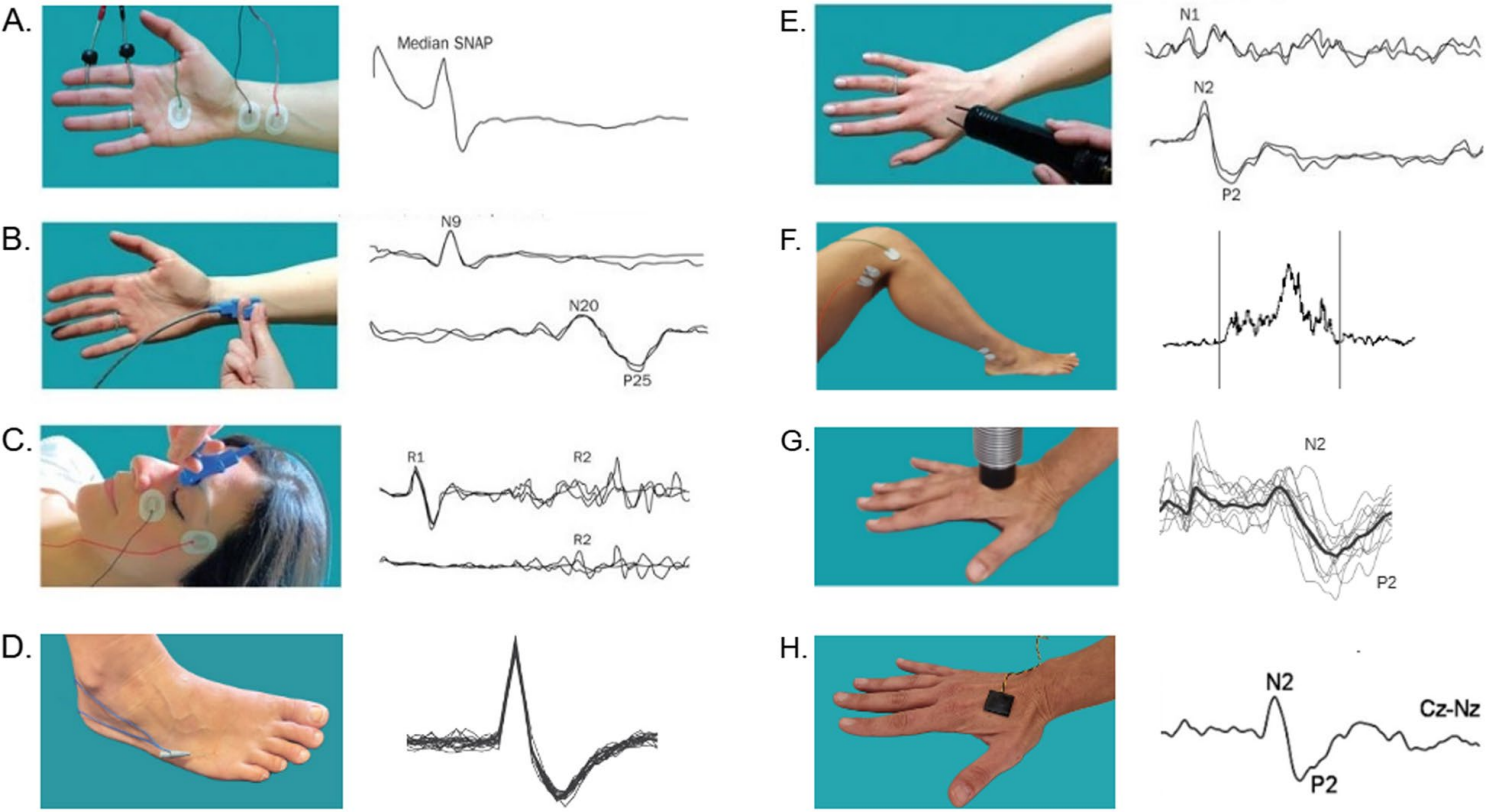
Neurophysiological diagnostic tools for investigating neuropathic pain mechanisms. **A** Nerve conduction study for the assessment of large myelinated Aβ fibres. **B** Somatosensory evoked potentials for the assessment of large myelinated Aβ fibres and dorsal column–medial lemniscus system at central level. **C** Trigeminal reflexes for the assessment of large myelinated Aβ fibres at trigeminal level. **D** Microneurography for the assessment of small myelinated Aδ and unmyelinated C fibres. **E** Laser evoked potentials for the assessment of small myelinated Aδ and unmyelinated C fibres. **F** RIII nociceptive flexion reflex for the assessment of small myelinated Aδ fibres. **G** Thermal evoked potentials for the assessment of small myelinated Aδ and unmyelinated C fibres. **H** Nociceptive evoked potentials through micropatterned surface electrode for the assessment of small myelinated Aδ fibres

Various neurophysiological methods have been developed to investigate large-myelinated non nociceptive fibres and small nociceptive fibres in patients with neuropathic pain [29]. The most commonly used neurophysiological tools today include standard neurophysiological techniques, microneurography, nociceptive reflexes, and nociceptive evoked potentials (Fig. 2).

In this argumentative text, we will show how these neurophysiological tools provide important information about the pathophysiological mechanisms underlying specific neuropathic pain symptoms.

### Neurophysiological techniques exploring large-myelinated fibres

Standard neurophysiological techniques, such as nerve conduction study (NCS) (Fig. 2A) and somatosensory evoked potentials (SEPs) (Fig. 2B), represent the reference standard techniques for investigating patients with peripheral and central nervous system diseases [16]. These techniques explore non-nociceptive system, namely large myelinated Aβ fibres in the peripheral nervous system, and in the dorsal column–medial lemniscus system at central level. NCS allows the assessment of amplitude and latency of nerve action potential and the conduction velocity of a peripheral nerve. It allows the diagnosis of both axonal and demyelinating peripheral neuropathies. SEPs reflect the electrical potentials generated in sensory pathways at peripheral, spinal, supraspinal, and cortical levels. Short-latency responses occur within the first 50 ms following a brief stimulus. Late responses, including middle and long-latency SEPs, also occur but have a broader range of normal variability, making them less practical for clinical use (for a detailed description of the different components, see [56].

The cervical spinal component N13 requires special consideration. It is mediated by non-nociceptive Aβ fibres, elicited by suprathreshold electrical stimulation of the median or ulnar nerve, and best recorded on the C6 spinous process referenced at the glottis [22]. Thanks to its peculiar features and its similarities with animals’ spinal potentials, suggesting a possible involvement of wide dynamic range neurons as a generator [8, 34], in recent times, the N13 has aroused interest as a possible readout of dorsal horn excitability changes occurring during central sensitization. Seminal papers proved the ability of this spinal component in detecting dorsal horn excitability induced by human models of secondary hyperalgesia [26, 48].

At trigeminal level, where standard neurophysiological techniques cannot be performed, trigeminal reflexes are commonly used (Fig. 2C). These reflexes, namely the blink reflex after stimulation of large myelinated fibres of the supraorbital nerve and the masseter inhibitory reflex after stimulation of large myelinated fibres of the infraorbital and mental nerves, allow the study of non-nociceptive trigeminal pathway [17].

Because most clinical and experimental studies showed that neuropathic pain is mainly related to nociceptive system damage, current knowledge postulates that the neurophysiological assessment of large non-nociceptive afferent fibres does not contribute to the diagnosis of neuropathic pain [30]. However, these standard techniques are still useful to demonstrate, locate, and quantify damage along the peripheral and central somatosensory system in patients with suspected neuropathic pain, given that most peripheral and central nervous system diseases manifesting with pain homogenously affect both non-nociceptive large-fibres and nociceptive small fibres [32].

### Neurophysiological techniques exploring small fibres

Microneurography is an invasive method to record action potentials from single afferent fibres in awake humans, by inserting needle electrodes into an accessible nerve (Fig. 2D). Though it could have the potential of disclosing abnormalities in small fibres functioning, it is considered an experimental tool, and its application in clinical practice is very limited [65].

The RIII flexion reflex (Fig. 2F), mediated by Aδ fibres, is elicited by electrical stimulation of a peripheral nerve, causing subclinical limb withdrawal at the spinal level [53]. While it can be evoked in both upper and lower limbs, most studies focus on the lower limbs due to simplicity and non-invasiveness. Typically, the sural nerve is stimulated with a train of five electrical pulses (0.1 ms each, 200 Hz), and EMG activity is recorded from the ipsilateral biceps femoris muscle [60, 81]. The reflex threshold closely correlates with the subjective pain threshold and serves as an objective measure in pain research at both spinal and supraspinal levels [61]. Recent reviews of the literature underlie its usefulness as a readout of spinal excitability [50].

During the last years, different evoked potential techniques for selective investigation of the nociceptive system have been devised [47]. Electrical stimulation of the intraepidermal nerve fibres has been proposed [28, 38], for the selective assessment of nociceptive system. There are different techniques devised for electrical stimulation and several authors have reported on their utility by showing reduced evoked potential amplitude in patients suffering from pain in comparison to healthy subjects [55, 77]. The specific device employed for electrical stimulation, however, may determine specificity. While the nociceptive specificity of the surface concentric electrode has been challenged [44, 59], intra-epidermal electrodes as well as micropatterned surface electrodes (Fig.2H) [28] were recently shown to have similar latencies as laser-evoked potentials in intra-cortical human recordings [36].

The most widely agreed techniques for the selective investigation of nociceptive system rely on radiant or contact heat stimuli (Fig. 2E–G), which selectively activate nociceptors, giving rise to laser evoked potentials and contact-heat evoked potentials [35, 73]. Laser and contact heat stimulation selectively activates Aδ and C nociceptors in the most superficial skin layers and evoked large amplitude scalp potentials. Although laser and contact heat stimuli activate both Aδ and C fibres, scalp potentials related to C-fibre activation can be obtained only with dedicated techniques that have not yet been standardised for clinical application [54]. Hence, the commonly studied LEPs are those related to Aδ-fibres activation. The scalp potentials related to contact-heat and laser stimulation consist of a lateralized component (N1), likely generated in the SII area and in the insular cortex bilaterally, and a vertex potential consisting of a N2–P2 complex [73]. Whereas the N2-LEP component probably reflects neuronal activity in anterior-mid insula and possibly the anterior cingulate cortex, the P2-LEP originates from the anterior cingulated cortex alone [31].

In peripheral and central nervous system diseases associated with nociceptive system damage, LEPs can be absent, reduced in amplitude, or delayed in latency. Although LEP recording is the most widely accepted method for investigating nociceptive fibres in patients with neuropathic pain, only a few studies have systematically evaluated the diagnostic accuracy of this technique (Truini et al., 2023 [68]). An additional limitation of LEP recordings is the influence of attention on the vertex N2-P2 complex, which may affect its diagnostic yield in identifying nociceptive system damage [69]).

The recent introduction of a novel device for the activation of cold receptors with very steep cooling ramps [20] has allowed the clinical assessment of patients complaining of cold pain (Fig. 2G). The technique consists of the cooling of the skin via a contact probe with a flat surface area of 160 mm^2^made up of 16 embedded micro-Peltier elements. The stimulation cools the skin with ramps of up to − 300 °C/s and allows the recording of scalp evoked potentials: a vertex component (N2-P2) and a lateralized component (N1) [49].

Each method for generating thermo-nociceptive evoked potentials has its pros and cons; all of them allow for a reproducible evaluation of the peripheral and central thermos-nociceptive pathway [78].

### Ongoing burning pain

In clinical practice, patients with neuropathic pain report ongoing burning pain in 65% of cases [4], variably associated with sensory loss such as thermal-pain hypoesthesia.

Previous studies have demonstrated that in patients with neuropathic pain due to peripheral and central nervous system diseases (such as postherpetic neuralgia, carpal tunnel syndrome, polyneuropathy, and multiple sclerosis), the severity of ongoing burning pain is inversely related to the amplitude of laser-evoked potentials [64, 70, 71, 75]. Although in some instances this relationship is only an indirect finding, it unquestionably indicates that ongoing burning pain is strongly associated with damage to the nociceptive system.

In certain conditions like postherpetic neuralgia and multiple sclerosis, burning pain is thought to result from a deafferentation mechanism [71, 76]. For instance, in patients with postherpetic neuralgia experiencing burning pain and severe sensory deficits, where dorsal root ganglion loss is assumed, the ongoing burning pain may originate from abnormal hyperactivity of second-order dorsal horn neurons deprived of primary afferent connections [30]. Accordingly, animal studies have shown that following peripheral nerve lesions causing significant loss of unmyelinated primary afferent function, many dorsal horn cells begin to fire spontaneously at high frequencies [52].

In multiple sclerosis patients, ongoing burning pain results from spinothalamic tract lesions, leading to deafferentation of thalamic nuclei [76]. Correspondingly, in rats, extracellular recordings from the ventro-posterolateral thalamic nucleus after experimental spinothalamic tract lesions have shown spontaneous and abnormal hyperactivity of neurons [37, 80].

Conversely, in patients with length-dependent axonal neuropathy, ongoing burning pain likely involves different pathophysiological mechanisms other than deafferentation. Microneurographic recordings of C-fibres in these patients have shown that ongoing pain, including the burning type, is associated with abnormally spontaneous active C-fibres [41, 62]. Since selective intraneural stimulation of C-fibres evokes burning pain in normal humans, it is reasonable to assume that the spontaneous activity in patients with peripheral neuropathies suffering from neuropathic pain is responsible for ongoing burning pain. This ‘hyperactivity’ involving nociceptive primary afferents is commonly referred to as peripheral sensitization [7, 10, 14, 30].

## Paroxysmal pain

In clinical practice, patients with neuropathic pain report paroxysmal pain in 57% of cases [4]. It is an intermittent pain that usually is not associated with any precursor and described as shooting, lancinating, jabbing or stabbing in nature.

Neurophysiological studies in animals support the involvement of large myelinated Aβ-fibre in paroxysmal pain. In models of peripheral neuropathy, large myelinated Aβ fibres are identified as the axonal population where early and predominantly ectopic activity develops post-injury [79, 82].

In patients with paroxysmal electrical shock-like pains due to peripheral and CNS diseases show damage to non-nociceptive large myelinated fibre pathways [74]. In cases of postherpetic neuralgia and carpal tunnel syndrome, this pain is linked to abnormalities in non-nociceptive Aβ-fibres [70, 71]. Specifically, the correlation between delayed blink reflex responses, slowed median-nerve sensory conduction, and paroxysmal electrical shock-like pain indicates focal Aβ-fibre demyelination. In multiple sclerosis, Lhermitte’s phenomenon, a classic electrical shock-like sensation, is associated with dorsal column abnormalities in somatosensory evoked potentials, while spinothalamic-mediated LEPs remain unaffected (Truini et al., 2012 [76]). This suggests that Lhermitte’s phenomenon results from demyelination in non-nociceptive dorsal columns, leaving nociceptive spinothalamic pathways largely intact.

The link between electrical shock-like pain and large myelinated fibre demyelination is supported by findings that trigeminal neuralgia, a condition typically causing such pain, results from focal demyelination of large myelinated Aβ fibres [19]. In classic trigeminal neuralgia (caused by vessels compressing the trigeminal root) and symptomatic trigeminal neuralgia (due to benign tumours compressing or stretching the root), mechanical compression damages large myelinated fibres, leading to demyelination [24]. Experimental studies in animals and humans show that nerve compression injures large myelinated Aβ fibres while sparing small myelinated and unmyelinated fibres [57]. Symptomatic trigeminal neuralgia from a pontine demyelinating plaque in multiple sclerosis also stems from primary afferent fibre demyelination [18]. Whether caused by multiple sclerosis or chronic compression from a blood vessel or tumour, Aβ fibre demyelination increases susceptibility to ectopic excitation and high-frequency discharges, resulting in paroxysmal pain [9].

More recently, the classic distinction, which posits that pain is exclusively conveyed by small-calibre Aδ and C fibres, has been questioned. A recent literature review has highlighted the existence of nociceptors in humans with very fast conducting afferents, within the conduction range of large diameter Aβ fibres [58]. These fibres could be directly involved in the genesis of paroxysmal pain.

Certain paroxysmal pains, such as the stabbing sensations in brachial plexus lesions or the burning paroxysms in paroxysmal extreme pain disorder, likely involve nociceptive fibre damage [1, 63]. Paroxysmal pain in brachial plexus avulsion responds to surgical lesions in the dorsal root entry zone, selectively damaging nociceptive second-order neurons while sparing dorsal columns [2]. Paroxysmal extreme pain disorder is caused by a gain-of-function mutation in SCN9A, the gene encoding the Na(v)1.7 sodium channel found in nociceptive fibres, leading to abnormal activity and paroxysmal pain [11].

Therefore, while ongoing burning pain always involves nociceptive fibre damage, paroxysmal pain can result from damage to either nociceptive or non-nociceptive pathways. Specifically, electric shock-like sensations are often due to damage to Aβ fibres.

## Provoked pain

In clinical practice, patients with neuropathic pain report provoked pain in 55% of cases [4].

Provoked pains are triggered by various stimuli. Hyperalgesia refers to increased pain from stimuli that normally cause pain (e.g., pinprick), while ‘allodynia’ describes pain from typically non-painful stimuli. Warm and cold allodynia refer to pain from mild temperature changes, and mechanical allodynia is termed ‘static’ if caused by slight pressure and ‘dynamic’ if caused by light moving touch. The most common type is dynamic mechanical allodynia, often experienced as pain from gentle brushing, affecting 18–54% of patients with neuropathic pain [4].

Clinical examples include thoracic postherpetic neuralgia patients feeling pain from shirt contact, peripheral neuropathy patients experiencing pain from bed sheet contact, and lateral medullary infarct patients reporting pain from light air currents on one side of the face [39].

Mechanical allodynia arises from lesions in pain pathways that cause central nociceptive neurons to respond to low-threshold Aβ afferents (Zimmermann, 2001 [83]). While both A- and C-fibres can develop abnormal sensitization after nerve injury, myelinated Aβ fibres are primarily involved in human mechanical dynamic allodynia [12]. Reaction times to allodynic stimuli do not match C-fibre conduction velocities, and differential nerve blocks show that allodynia disappears with the loss of Aβ-driven tactile sensation, while Aδ- and C-fibre functions remain intact [45]. In patients with neuropathic pain, blocking A-fibres eliminates allodynia but not ongoing burning pain from C-afferents [43].

A continuum between mechanical allodynia and non-painful dysesthesia during progressive Aβ fibre block indicates that the intensity of the sensation depends on the number of Aβ mechanoreceptive fibres accessing central nociceptive systems [45]. Therefore, Aβ fibres are the main contributors to dynamic mechanical allodynia following peripheral lesions. Animal model results support this, showing that Aβ fibres are mainly responsible for ectopic response generation and their increased excitability after nerve lesions correlates with allodynic-like behaviour development [25]. The activation of nociceptive signals in the CNS is evident in the burning, pricking, or sore sensations of mechanical allodynia. Transcutaneous or intraneural stimuli can evoke burning pain and trigger nociceptive flexion reflexes, even at intensities that produce non-painful sensations in normal skin ([67]; Garcia Larrea and Mauguiere, 1990 [33]). Reaction times to allodynic stimuli (around 450 ms) suggest involvement of both rapid peripheral and slow central conducting fibres.

Nociceptive activation by Aβ fibres likely involves nociceptive-specific projecting neurons rather than wide-dynamic range (WDR) cells, as descending noxious inhibitory controls, which inhibit WDR cells, do not affect mechanical allodynia [46]. Consequently, mechanical allodynia requires at least partial functionality of the ascending spinothalamic system. Studies show that patients with partially preserved nociceptive pathway conduction, as indicated by LEPs, have a higher likelihood of developing allodynia [72] zim.

Provoked pain is common complaint also in patients with central nervous system diseases. For instance, neuropathic pain from thalamic lesions often includes cold allodynia, indicating damage to the cold sensory system [40]. The ‘thalamic disinhibition hypothesis’ [15] suggests that an imbalance between cold-afferent and thermal-pain pathways causes this central post-stroke pain. A previous study showed that in a patient with cold-allodynia related to a thalamic lesion, cold evoked potential recording disclosed the partial preservation of the nociceptive pathway in association with the impairment of the cold-pathway, indirectly confirming the ‘thalamic disinhibition hypothesis’ [49].

Several chronic pain conditions are associated with diffuse hyperalgesia, i.e. increased responsiveness to pinprick stimuli. These conditions include fibromyalgia syndrome, complex regional pain syndrome type 1, ‘nonspecific’ chronic low- back pain, whiplash syndrome, irritable bowel syndrome, painful bladder syndrome, and chronic migraine [3]. The diffuse hyperalgesia, a frequent finding in patients with chronic pain, is thought to be subtended by central sensitization, which in turn likely results from synaptic plasticity phenomena of synaptic strength long-term potentiation (LTP) in the central nervous system [42]. While the direct recording of central nociceptive neurons in the spinal cord cannot be performed, some neurophysiological tools can be used to indirectly detect spinal hyperexcitability phenomena in humans, including the RIII reflex and the N13 component of the somatosensory evoked potentials.

The RIII has been used to study the pathophysiology of clinical syndromes characterized by chronic pain, such as thalamic pain syndrome, migraine, and fibromyalgia. It has been found to be consistently hyperexcitable in painful conditions underlie by central sensitization, in terms of decreased reflex and pain thresholds ([6, 13, 23, 51]).

Previous studies on the N13-SEP component provided evidence for the involvement of wide dynamic range neurons in the generation of this measure [27] and its modulation by experimental pain models of central sensitization [26, 48]. Though the ability of the N13 component to detect spinal hyperexcitability is still in its infancy and primarily inferred from studies on healthy populations with experimental pain models, a previous paper on the neuroplastic changes related to cervical radicular pain described an increased N13 component from stimulation of the painful side [66], suggesting that this component might be an interesting readout of hyperalgesia.

## Conclusions

Neuropathic pain arises from damage or disease affecting the somatosensory system. Dedicated neurophysiological tools can provide important, complementary insights and help disentangle the pathophysiological mechanisms underlying various neuropathic pain symptoms. Identifying these specific mechanisms may guide drug selection and significantly improve treatment success rates.

## Data Availability

No datasets were generated or analysed during the current study.

## References

[1] Aichaoui F, Mertens P, Sindou M. Dorsal root entry zone lesioning for pain after brachial plexus avulsion: results with special emphasis on differential effects on the paroxysmal versus the continuous components. A prospective study in a 29-patient consecutive series. Pain. 2011;152:1923–1930. 10.1016/j.pain.2011.03.037. 10.1016/j.pain.2011.03.037 21549506

[2] Ali M, Saitoh Y, Oshino S, Hosomi K, Kishima H, Morris S, Shibata M, Yoshimine T (2011) Differential efficacy of electric motor cortex stimulation and lesioning of the dorsal root entry zone for continuous vs paroxysmal pain after brachial plexus avulsion. Neurosurgery 68:1252–1257. 10.1227/NEU.0b013e31820c04a9 21307799 10.1227/NEU.0b013e31820c04a9

[3] Arendt-Nielsen L, Morlion B, Perrot S, Dahan A, Dickenson A, Kress HG, Wells C, Bouhassira D, Mohr DA (2018) Assessment and manifestation of central sensitisation across different chronic pain conditions. Eur J Pain 22(2):216–241. 10.1002/ejp.1140 29105941 10.1002/ejp.1140

[4] Attal N, Fermanian C, Fermanian J, Lanteri-Minet M, Alchaar H, Bouhassira D (2008) Neuropathic pain: are there distinct subtypes depending on the aetiology or anatomical lesion? Pain 138(2):343–353. 10.1016/j.pain.2008.01.006 18289791 10.1016/j.pain.2008.01.006

[5] Attal N, de Andrade DC, Adam F, Ranoux D, Teixeira MJ, Galhardoni R, Raicher I, Üçeyler N, Sommer C, Bouhassira D (2016) Safety and efficacy of repeated injections of botulinum toxin A in peripheral neuropathic pain (BOTNEP): a randomised, double-blind, placebo-controlled trial. Lancet Neurol 15(6):555–565. 10.1016/S1474-4422(16)00017-X 26947719 10.1016/S1474-4422(16)00017-X

[6] Banic B, Petersen-Felix S, Andersen OK, Radanov BP, Villiger PM, Arendt-Nielsen L, Curatolo M (2004) Evidence for spinal cord hypersensitivity in chronic pain after whiplash injury and in fibromyalgia. Pain 107:7–15. 10.1016/j.pain.2003.05.001 14715383 10.1016/j.pain.2003.05.001

[7] Baron R, Binder A, Wasner G (2010) Neuropathic pain: diagnosis, pathophysiological mechanisms, and treatment. Lancet Neurol 9(8):807–819. 10.1016/S1474-4422(10)70143-5 20650402 10.1016/S1474-4422(10)70143-5

[8] Beall JE, Applebaum AE, Foreman RD, Willis WD (1977) Spinal cord potentials evoked by cutaneous afferents in the monkey. J Neurophysiol 40(2):199–211. 10.1152/jn.1977.40.2.199 403249 10.1152/jn.1977.40.2.199

[9] Burchiel KJ (1980) Ectopic impulse generation in focally demyelinated trigeminal nerve. Exp Neurol 69:423–429. 10.1016/0014-4886(80)90225-3 7409056 10.1016/0014-4886(80)90225-3

[10] Campbell JN, Meyer RA (2006) Mechanisms of neuropathic pain. Neuron 52:77–92. 10.1016/j.neuron.2006.09.021 17015228 10.1016/j.neuron.2006.09.021 PMC1810425

[11] Choi JS, Boralevi F, Brissaud O, Sánchez-Martín J, Te Morsche RH, Dib-Hajj SD, Drenth JP, Waxman SG (2011) Paroxysmal extreme pain disorder: a molecular lesion of peripheral neurons. Nat Rev Neurol 7:51–55. 10.1038/nrneurol.2010.162 21079636 10.1038/nrneurol.2010.162

[12] Cline MA, Ochoa J, Torebjörk HE (1989) Chronic hyperalgesia and skin warming caused by sensitized C nociceptors. Brain 112:621–647. 10.1093/brain/112.3.621 2731024 10.1093/brain/112.3.621

[13] Coffin B, Bouhassira D, Sabaté JM, Barbe L, Jian R (2004) Alteration of the spinal modulation of nociceptive processing in patients with irritable bowel syndrome. Gut 53:1465–1470. 10.1136/gut.2003.031310 15361496 10.1136/gut.2003.031310 PMC1774236

[14] Costigan M, Scholz J, Woolf CJ (2009) Neuropathic pain: a maladaptive response of the nervous system to damage. Annu Rev Neurosci 32:1–32 19400724 10.1146/annurev.neuro.051508.135531 PMC2768555

[15] Craig AD (1998) A new version of the thalamic disinhibition hypothesis of central pain. Pain Forum 7:1–14

[16] Cruccu G, Aminoff MJ, Curio G, Guerit JM, Kakigi R, Mauguiere F, Rossini PM, Treede RD, Garcia-Larrea L (2008) Recommendations for the clinical use of somatosensory-evoked potentials. Clin Neurophysiol 119(8):1705–1719. 10.1016/j.clinph.2008.03.016 18486546 10.1016/j.clinph.2008.03.016

[17] Cruccu G, Deuschl G (2000) The clinical use of brainstem reflexes and hand-muscle reflexes. Clin Neurophysiol 111(3):371–387. 10.1016/s1388-2457(99)00291-6 10699396 10.1016/s1388-2457(99)00291-6

[18] Cruccu G, Biasiotta A, Di Rezze S, Fiorelli M, Galeotti F, Innocenti P, Mameli S, Millefiorini E, Truini A (2009) Trigeminal neuralgia and pain related to multiple sclerosis. Pain 143:186–191. 10.1016/j.pain.2008.12.026 19171430 10.1016/j.pain.2008.12.026

[19] Cruccu G, Di Stefano G, Truini A (2020) Trigeminal Neuralgia. N Engl J Med 383(8):754–762. 10.1056/NEJMra1914484 32813951 10.1056/NEJMra1914484

[20] De Keyser R, van den Broeke EN, Courtin A, Dufour A, Mouraux A (2018) Event-related brain potentials elicited by high-speed cooling of the skin: a robust and non-painful method to assess the spinothalamic system in humans. Clin Neurophysiol 129(5):1011–1019. 10.1016/j.clinph.2018.02.123 29567583 10.1016/j.clinph.2018.02.123 PMC6398569

[21] Demant DT, Lund K, Vollert J, Maier C, Segerdahl M, Finnerup NB, Jensen TS, Sindrup SH (2014) The effect of oxcarbazepine in peripheral neuropathic pain depends on pain phenotype: a randomised, double-blind, placebo-controlled phenotype-stratified study. Pain 155(11):2263–2273. 10.1016/j.pain.2014.08.014 25139589 10.1016/j.pain.2014.08.014

[22] Desmedt JE, Cheron G (1980) Central somatosensory conduction in man: neural generators and interpeak latencies of the far-field components recorded from neck and right or left scalp and earlobes. Electroencephalogr Clin Neurophysiol 50(5–6):382–403. 10.1016/0013-4694(80)90006-1 6160982 10.1016/0013-4694(80)90006-1

[23] Desmeules JA, Cedraschi C, Rapiti E, Baumgartner E, Finckh A, Cohen P, Dayer P, Vischer TL (2003) Neurophysiologic evidence for a central sensitization in patients with fibromyalgia. Arthritis Rheum 48(5):1420–1429. 10.1002/art.10893 12746916 10.1002/art.10893

[24] Devor M, Amir R, Rappaport ZH (2002) Pathophysiology of trigeminal neuralgia: the ignition hypothesis. Clin J Pain 18(1):4–13. 10.1097/00002508-200201000-00002 11803297 10.1097/00002508-200201000-00002

[25] Devor M (2009) Ectopic discharge in Aβ afferents as a source of neuropathic pain. Exp Brain Res 196:115–128. 10.1007/s00221-009-1724-6 19242687 10.1007/s00221-009-1724-6

[26] Di Lionardo A, Di Stefano G, Leone C, Di Pietro G, Sgro E, Malara E, Cosentino C et al (2021) Modulation of the N13 component of the somatosensory evoked potentials in an experimental model of central sensitization in humans. Sci Rep 21(11):20838. 10.1038/s41598-021-00313-7 10.1038/s41598-021-00313-7 PMC8531029 34675309

[27] Di Pietro G, Di Stefano G, Leone C, Di Lionardo A, Sgrò E, Blockeel AJ, Caspani O et al (2021) The N13 spinal component of somatosensory evoked potentials is modulated by heterotopic noxious conditioning stimulation suggesting an involvement of spinal wide dynamic range neurons. Neurophysiol Clin 51(6):517–523. 10.1016/j.neucli.2021.09.001 34756635 10.1016/j.neucli.2021.09.001

[28] Di Stefano G, Di Lionardo A, La Cesa S, Di Pietro G, Fasolino A, Galosi E, Leone C et al (2020) The new micropatterned interdigitated electrode for selective assessment of the nociceptive system. Eur J Pain 24(5):956–966. 10.1002/ejp.1545 32064700 10.1002/ejp.1545

[29] Di Stefano G, La Cesa S, Biasiotta A, Leone C, Pepe A, Cruccu G, Truini A (2012) Laboratory tools for assessing neuropathic pain. Neurol Sci 33(Suppl 1):S5-7. 10.1007/s10072-012-1033-x 22644160 10.1007/s10072-012-1033-x

[30] Fields HL, Rowbotham M, Baron R (1998) Postherpetic neuralgia: irritable nociceptors and deafferentation. Neurobiol Dis 5:209–227 9848092 10.1006/nbdi.1998.0204

[31] Garcia-Larrea L, Frot M, Valeriani M (2003) Brain generators of laser-evoked potentials: from dipoles to functional significance. Neurophysiol Clin 33(6):279–292. 10.1016/j.neucli.2003.10.008 14678842 10.1016/j.neucli.2003.10.008

[32] Garcia-Larrea L, Hagiwara K. Electrophysiology in diagnosis and management of neuropathic pain. Rev Neurol (Paris). 2019;175(1–2):26–37. 10.1016/j.neurol.2018.09.015 10.1016/j.neurol.2018.09.015 30482566

[33] García-Larrea L, Mauguière F (1990) Electrophysiological assessment of nociception in normals and patients: the use of nociceptive reflexes. Electroencephalogr Clin Neurophysiol Suppl 41:102–18. 10.1016/b978-0-444-81352-7.50013-3 10.1016/b978-0-444-81352-7.50013-3 2289417

[34] Gasser HS, Graham HT (1933) Potentials produced in the spinal cord by stimulation of dorsal roots. Am J Physiol 103(2):303–320. 10.1152/ajplegacy.1933.103.2.303

[35] Granovsky Y, Anand P, Nakae A, Nascimento O, Smith B, Sprecher E, Valls-Solé J (2016) Normative data for Aδ contact heat evoked potentials in adult population: a multicenter study. Pain 157(5):1156–1163. 10.1097/j.pain.0000000000000495 26907092 10.1097/j.pain.0000000000000495

[36] Hagiwara K, Perchet C, Frot M, Bastuji H, Garcia-Larrea L (2018) Insular-limbic dissociation to intra-epidermal electrical Aδ activation: a comparative study with thermo-nociceptive laser stimulation. Eur J Neurosci 48(10):3186–3198. 10.1111/ejn.14146 30203624 10.1111/ejn.14146

[37] Hains BC, Saab CY, Waxman SG. Changes in electrophysiological properties and sodium channel Nav1.3 expression in thalamic neurons after spinal cord injury. Brain. 2005;128:2359–2371. 10.1093/brain/awh623. 10.1093/brain/awh623 16109750

[38] Inui K, Tran TD, Hoshiyama M, Kakigi R (2002) Preferential stimulation of Aδ fibers by intra-epidermal needle electrode in humans. Pain 96(3):247–252. 10.1016/S0304-3959(01)00453-5 11972996 10.1016/S0304-3959(01)00453-5

[39] Jensen TS, Finnerup NB (2014) Allodynia and hyperalgesia in neuropathic pain: clinical manifestations and mechanisms. Lancet Neurol 13:924–935. 10.1016/S1474-4422(14)70102-4 25142459 10.1016/S1474-4422(14)70102-4

[40] Kim JH, Greenspan JD, Coghill RC, Ohara S, Lenz FA (2007) Lesions limited to the human thalamic principal somatosensory nucleus (ventral caudal) are associated with loss of cold sensations and central pain. J Neurosci 27:4995–5004. 10.1523/JNEUROSCI.0716-07.2007 17475808 10.1523/JNEUROSCI.0716-07.2007 PMC6672095

[41] Kleggetveit IP, Namer B, Schmidt R, Helås T, Rückel M, Ørstavik K, Schmelz M, Jørum E (2012) High spontaneous activity of C-nociceptors in painful polyneuropathy. Pain 153:2040–2047. 10.1016/j.pain.2012.05.017 22986070 10.1016/j.pain.2012.05.017

[42] Klein T, Stahn S, Magerl W, Treede RD (2008) The role of heterosynaptic facilitation in long-term potentiation (LTP) of human pain sensation. Pain 139(3):507–519. 10.1016/j.pain.2008.06.001 18602755 10.1016/j.pain.2008.06.001

[43] Koltzenburg M, Torebjork HE, Wahren LK (1994) Nociceptor modulated central sensitization causes mechanical hyperalgesia in acute chemogenic and chronic neuropathic pain. Brain 117:579–591 8032867 10.1093/brain/117.3.579

[44] La Cesa S, Di Stefano G, Leone C, Pepe A, Galosi E, Alu F, Fasolino A, Cruccu G, Valeriani M, Truini A (2018) Skin denervation does not alter cortical potentials to surface concentric electrode stimulation: a comparison with laser evoked potentials and contact heat evoked potentials. Eur J Pain 22(1):161–169. 10.1002/ejp.1112 28898491 10.1002/ejp.1112

[45] Landerholm ÅH, Hansson PT (2011) Mechanisms of dynamic mechanical allodynia and dysesthesia in patients with peripheral and central neuropathic pain. Eur J Pain 15:498–503 21094619 10.1016/j.ejpain.2010.10.003

[46] Le Bars D, Dickenson AH, Besson JM. Diffuse noxious inhibitory controls (DNIC). II. Lack of effect on non-convergent neurones, supraspinal involvement and theoretical implications. Pain. 1979;6:305–327. 10.1016/0304-3959(79)90050-2 460936

[47] Lefaucheur JP (2019) Clinical neurophysiology of pain. Handb Clin Neurol 161:121–148. 10.1016/B978-0-444-64142-7.00045-X 31307596 10.1016/B978-0-444-64142-7.00045-X

[48] Leone C, Di Pietro G, Salman Y, Galosi E, Di Stefano G, Caspani O, Garcia-Larrea L, Mouraux A, Treede RD, Truini A (2023) Modulation of the spinal N13 SEP component by high- and low-frequency electrical stimulation. Exp pain models matter Clin Neurophysiol 156:28–37. 10.1016/j.clinph.2023.08.022 10.1016/j.clinph.2023.08.022 37856896

[49] Leone C, Dufour A, Di Stefano G, Fasolino A, Di Lionardo A, La Cesa S, Galosi E et al (2019) Cooling the skin for assessing small-fibre function. Pain 160(9):1967–1975. 10.1097/j.pain.0000000000001584 30985621 10.1097/j.pain.0000000000001584

[50] Leone CM, Lenoir C, van den Broeke EN (2024). Eur J Pain. 10.1002/ejp.4733Assessingsignsofcentralsensitization:acriticalreviewofphysiologicalmeasuresinexperimentallyinducedsecondaryhyperalgesia39315535

[51] Leroux A, Bélanger M, Boucher JP (1995) Pain effect on monosynaptic and polysynaptic reflex inhibition. Arch Phys Med Rehabil 76(6):576–582 7763159 10.1016/s0003-9993(95)80514-1

[52] Liu CN, Wall PD, Ben-Dor E, Michaelis M, Amir R, Devor M (2000) Tactile allodynia in the absence of C-fiber activation: altered firing properties of DRG neurons following spinal nerve injury. Pain 85:503–521. 10.1016/S0304-3959(00)00251-7 10781925 10.1016/S0304-3959(00)00251-7

[53] Meinck HM, Piesiur-Strehlow B, Koehler W (1981) Some principles of flexor reflex generation in human leg muscles. Electroencephalogr Clin Neurophysiol 52:140–150 6167423 10.1016/0013-4694(81)90161-9

[54] Mouraux A, Plaghki L. Chapter 27: Are the processes reflected by late and ultra-late laser evoked potentials specific of nociception? In: Supplements to Clinical Neurophysiology. Elsevier; 2006. p. 197–204. 10.1016/S1567-424X(09)70031-5. 10.1016/s1567-424x(09)70031-5 16893112

[55] Mueller D, Obermann M, Koeppen S, Kavuk I, Yoon MS, Sack F, Diener HC, Kaube H, Katsarava Z (2010) Electrically evoked nociceptive potentials for early detection of diabetic small-fiber neuropathy. Eur J Neurol 17(6):834–841. 10.1111/j.1468-1331.2009.02938.x 20192984 10.1111/j.1468-1331.2009.02938.x

[56] Nuwer MR, Aminoff M, Desmedt J, Eisen AA, Goodin D, Matsuoka S, Mauguière F, Shibasaki H, Sutherling W, Vibert JF. IFCN recommended standards for short latency somatosensory evoked potentials. Report of an IFCN committee. International Federation of Clinical Neurophysiology. Electroencephalogr Clin Neurophysiol. 1994;91(1):6–11. 10.1016/0013-4694(94)90012-4 10.1016/0013-4694(94)90012-4 7517845

[57] Ochoa J, Fowler TJ, Gilliatt RW (1972) Anatomical changes in peripheral nerves compressed by a pneumatic tourniquet. J Anat 113:433–455 4197303 PMC1271414

[58] Olausson H, Marshall A, Nagi SS, Cole J (2024) Slow touch and ultrafast pain fibres: revisiting peripheral nerve classification. Clin Neurophysiol 163:255–262. 10.1016/j.clinph.2024.04.008 38704307 10.1016/j.clinph.2024.04.008

[59] Perchet C, Frot M, Charmarty A, Flores C, Mazza S, Magnin M, Garcia-Larrea L (2012) Do we activate specifically somatosensory thin fibres with the concentric planar electrode? A scalp and intracranial EEG study. Pain 153(6):1244–1252. 10.1016/j.pain.2012.03.004 22497800 10.1016/j.pain.2012.03.004

[60] Sandrini G, Arrigo A, Bono G, Nappi G (1993) The nociceptive flexion reflex as a tool for exploring pain control systems in headache and other pain syndromes. Cephalalgia 13(1):21–27. 10.1046/j.1468-2982.1993.1301021.x 8448783 10.1046/j.1468-2982.1993.1301021.x

[61] Sandrini G, Serrao M, Rossi P, Romaniello A, Cruccu G, Willer JC (2005) The lower limb flexion reflex in humans. Prog Neurobiol 77(6):353–395. 10.1016/j.pneurobio.2005.11.003 16386347 10.1016/j.pneurobio.2005.11.003

[62] Serra J (2012) Microneurography: towards a biomarker of spontaneous pain. Pain 153:1989–1990 22820023 10.1016/j.pain.2012.07.008

[63] Sindou M (2011) Surgery in the DREZ for refractory neuropathic pain after spinal cord/cauda equina injury. World Neurosurg 75:447–448. 10.1016/j.wneu.2011.01.034 21600493 10.1016/j.wneu.2011.01.034

[64] Spiegel J, Hansen C, Baumgärtner U, Hopf HC, Treede RD (2003) Sensitivity of laser-evoked potentials versus somatosensory evoked potentials in patients with multiple sclerosis. Clin Neurophysiol 114(6):992–1002. 10.1016/s1388-2457(03)00069-5 12804667 10.1016/s1388-2457(03)00069-5

[65] Themistocleous AC, Ramirez JD, Serra J, Bennett DL (2014) The clinical approach to small fibre neuropathy and painful channelopathy. Pract Neurol 14(6):368–379. 10.1136/practneurol-2013-000758 24778270 10.1136/practneurol-2013-000758 PMC4251302

[66] Tinazzi M, Fiaschi A, Rosso T, Faccioli F, Grosslercher J, Aglioti SM (2000) Neuroplastic changes related to pain occur at multiple levels of the human somatosensory system: a somatosensory-evoked potentials study in patients with cervical radicular pain. J Neurosci 20(24):9277–9283. 10.1523/JNEUROSCI.20-24-09277.2000 11125006 10.1523/JNEUROSCI.20-24-09277.2000 PMC6773009

[67] Torebjork HE, Lundberg LE, LaMotte RH (1992) Central changes in processing of mechanoreceptive input in capsaicin-induced secondary hyperalgesia in humans. J Physiol 448:765–780 1593489 10.1113/jphysiol.1992.sp019069 PMC1176227

[68] Truini A, Aleksovska K, Anderson CC, Attal N, Baron R, Bennett DL, Bouhassira D, Cruccu G, Eisenberg E, Enax-Krumova E, Davis KD, Di Stefano G, Finnerup NB, Garcia-Larrea L, Hanafi I, Haroutounian S, Karlsson P, Rakusa M, Rice ASC, Sachau J, Smith BH, Sommer C, Tölle T, Valls-Solé J, Veluchamy A (2023) Joint European Academy of Neurology-European Pain Federation-Neuropathic Pain Special Interest Group of the International Association for the Study of Pain guidelines on neuropathic pain assessment. Eur J Neurol 30(8):2177–2196. 10.1111/ene.15831 10.1111/ene.15831 37253688

[69] Truini A, Galeotti F, Cruccu G, Garcia-Larrea L (2007) Inhibition of cortical responses to Adelta inputs by a preceding C-related response: testing the “first come, first served” hypothesis of cortical laser evoked potentials. Pain 131(3):341–347. 10.1016/j.pain.2007.06.023 17709206 10.1016/j.pain.2007.06.023

[70] Truini A, Padua L, Biasiotta A, Caliandro P, Pazzaglia C, Galeotti F, Inghilleri M, Cruccu G (2009) Differential involvement of A-delta and A-beta fibres in neuropathic pain related to carpal tunnel syndrome. Pain 145:105–109. 10.1016/j.pain.2008.08.018 19535205 10.1016/j.pain.2009.05.023

[71] Truini A, Galeotti F, Haanpaa M, Zucchi R, Albanesi A, Biasiotta A, Gatti A, Cruccu G (2008) Pathophysiology of pain in postherpetic neuralgia: a clinical and neurophysiological study. Pain 140:405–410. 10.1016/j.pain.2008.08.018 18954941 10.1016/j.pain.2008.08.018

[72] Truini A, Biasiotta A, Di Stefano G, La Cesa S, Leone C, Cartoni C, Leonetti F, Casato M, Pergolini M, Petrucci MT, Cruccu G (2013) Peripheral nociceptor sensitization mediates allodynia in patients with distal symmetric polyneuropathy. J Neurol 260:761–766. 10.1007/s00415-012-6698-9 23052607 10.1007/s00415-012-6698-9

[73] Truini A, Galeotti F, Romaniello A, Virtuoso M, Iannetti GD, Cruccu G (2005) Laser-evoked potentials: normative values. Clin Neurophysiol 116(4):821–826. 10.1016/j.clinph.2004.10.004 15792891 10.1016/j.clinph.2004.10.004

[74] Truini A, Garcia-Larrea L, Cruccu G (2013) Reappraising neuropathic pain in humans–how symptoms help disclose mechanisms. Nat Rev Neurol 9(10):572–582. 10.1038/nrneurol.2013.180 24018479 10.1038/nrneurol.2013.180

[75] Truini A, Romaniello A, Galeotti F, Iannetti GD, Cruccu G (2004) Laser evoked potentials for assessing sensory neuropathy in human patients. Neurosci Lett 361(1–3):25–28. 10.1016/j.neulet.2003.12.008 15135884 10.1016/j.neulet.2003.12.008

[76] Truini A, Galeotti F, La Cesa S, Di Rezze S, Biasiotta A, Di Stefano G, Tinelli E, Millefiorini E, Gatti A, Cruccu G (2012) Mechanisms of pain in multiple sclerosis: a combined clinical and neurophysiological study. Pain 153(10):2048–2054. 10.1016/j.pain.2012.05.024 22789132 10.1016/j.pain.2012.05.024

[77] Üçeyler N, Zeller D, Kahn AK, Kewenig S, Kittel-Schneider S, Schmid A, Casanova-Molla J, Reiners K, Sommer C (2013) Small fibre pathology in patients with fibromyalgia syndrome. Brain 136(6):1857–1867. 10.1093/brain/awt053 23474848 10.1093/brain/awt053

[78] Verdugo RJ, Matamala JM, Inui K, Kakigi R, Valls-Solé J, Hansson P, Nilsen KB, Lombardi R, Lauria G, Petropoulos IN, Malik RA, Treede RD, Baumgärtner U, Jara PA, Campero M (2022) Review of techniques useful for the assessment of sensory small fiber neuropathies: report from an IFCN expert group. Clin Neurophysiol 136:13–38. 10.1016/j.clinph.2022.01.002 35131635 10.1016/j.clinph.2022.01.002

[79] Wallace VC, Cottrell DF, Brophy PJ, Fleetwood-Walker SM (2003) Focal lysolecithin-induced demyelination of peripheral afferents results in neuropathic pain behavior that is attenuated by cannabinoids. J Neurosci 15:3221–3233. 10.1523/JNEUROSCI.23-08-03221.2003 10.1523/JNEUROSCI.23-08-03221.2003 PMC6742302 12716929

[80] Waxman SG, Hains BC (2006) Fire and phantoms after spinal cord injury: Na+ channels and central pain. Trends Neurosci 29:207–215. 10.1016/j.tins.2006.02.003 16494954 10.1016/j.tins.2006.02.003

[81] Willer JC (1977) Comparative study of perceived pain and nociceptive flexion reflex in man. Pain 3:69–80 876668 10.1016/0304-3959(77)90036-7

[82] Zhu YF, Henry JL (2012) Excitability of Aβ sensory neurons is altered in an animal model of peripheral neuropathy. BMC Neurosci 13:15. 10.1186/1471-2202-13-15 22289651 10.1186/1471-2202-13-15 PMC3292996

[83] Zimmermann M (2001) Pathobiology of neuropathic pain. Eur J Pharmacol 429(1-3):23–37. 10.1016/s0014-2999(01)01303-6 10.1016/s0014-2999(01)01303-6 11698024

